# Preferences for pitch sequence complexity are consistent with an inverted U-shape in humans and parrots

**DOI:** 10.1098/rsos.252399

**Published:** 2026-10-21

**Authors:** Oliver T. Bellmann, Marisa Hoeschele, Melina Witt, Eva Shair Ali, Pui Chu, Roberta Bianco

**Affiliations:** ^1^ Acoustics Research Institute, Austrian Academy of Sciences, Vienna, Austria; ^2^ Department of Behavioral and Cognitive Biology, University of Vienna, Vienna, Austria; ^3^ Vienna Doctoral School in Cognition, Behaviour and Neuroscience, University of Vienna, Vienna, Austria; ^4^ Department of Translational Research and New Technologies in Medicine and Surgery, University of Pisa, Pisa, Italy

**Keywords:** inverted U-shape, preference, complexity, auditory scene analysis, pattern detection, budgerigars, cross-species comparison, prediction by partial matching (PPM)

## Abstract

A well-established inverted U-shape relationship links stimulus complexity and human music preference, with preferences peaking at intermediate levels. We investigated whether preference for intermediate complexity is unique to humans or shared with other species. We compared the acoustic preferences of humans and budgerigars, a species known for their vocal learning and pattern recognition abilities, using a three-option place preference paradigm. Participants were presented with choices between stimuli of different, computationally defined complexity: pitch sequences with tones arranged either randomly (high complexity), repeating patterns with changes (medium) or fully repetitive patterns (low). Our results demonstrate that, without specific goals or external rewards, both humans and budgerigars spontaneously exhibit a nonlinear (intermediate) preference profile, indicating a pattern consistent with an inverted U-shape relationship. Because a U-shape relationship may reflect a strategy to seek informative sources maximally useful for learning—that is, for building and refining internal models of the environment—this preference in the acoustic domain may support vocal learning. However, further testing could determine whether it also exists in vocal non-learning species. Because vertebrates show inverted U-shape responses in a variety of non-auditory contexts, our findings may stem from general processes, but potentially depend on vocal learning to translate to acoustic contexts.

## 1. Introduction

A well-established inverted U-shape relationship exists between stimulus complexity (or related factors such as stress or arousal) and preference (‘the *U*’ [1]). That is, humans prefer stimuli with medium complexity compared to those with low or high complexity, with preferences changing predictably based on levels of cognitive effort and arousal or interest ([2,3]; for a review, see [4]). This might be because complex stimuli increase processing difficulty, demanding higher arousal to process [5]. Crucially, this preference function is hypothesized to serve a biological purpose: by favouring intermediate complexity, organisms maximize information gain while avoiding the cognitive stress of overload or the boredom of under-stimulation, directly linking preference to learnability [1,6].

While less is known about aesthetic preferences in non-human animals, this underlying link between intermediate challenge and optimal learning appears across species (e.g. [7,8]). In zebra finches, the quality of song imitation follows an inverted U-pattern relative to the number of song exemplars heard; both very low and very high exposure levels led to poorer imitation compared to an intermediate level [9], indicating that there is an optimal level of information for effective song learning. This suggests that *the U* in this context generally reflects a link between optimal performance and moderate levels of challenge, unlike other cognitive processes that follow asymptotic trajectories (e.g. working memory recall, speed of processing, verbal fluency, response inhibition) [10–12]. Accordingly, *the U* was hypothesized to be grounded in mechanisms guiding organisms to stimuli maximally useful for learning [1,6].

Learning plays a crucial role in many animal acoustic communication systems. Music and bird song, for instance, rely on the repeated social transmission of acoustic patterns [13–16]. Social learning needed for cultural transmission, here, occurs both horizontally (between peers) and vertically (across generations) [17]. Much like birdsong, which is shaped and transmitted through social learning [18], human musical traditions also rely on interaction and imitation [19], with repeated transmission leading to increasingly structured and more optimally learnable musical information (e.g. [20]). However, with each instance of transmission, acoustic patterns also must pass a ‘learner bottleneck’ [21]. That is, overly complex sequences or configurations are hard for receivers to learn and reproduce, hampering communicative efficiency. Similarly, information that is too simple, such as a pattern highly similar to knowledge already available to or held by an organism, offers little learning opportunity, as research in the visual domain suggests [6]. The *U* indeed plays an important role in music, which is transmitted via social learning and often relies on repetitive patterns [22–31]. For example, research using iterated learning paradigms shows that initially random sequences of note onsets (i.e. rhythm) [32–34] or pitches (i.e. melodies) [20,35,36] gradually condense into sets of discrete and small-integer-ratio elements and get less random. This process, improving signal quality and ultimately facilitating learnability, highlights how music is shaped by both cultural evolution and cognitive biases. Indeed, humans prefer music with a balance of predictability and novelty, avoiding overly simple and overly complex stimuli during both perception and cultural transmission ([37–45]; for a review, see [4]).

This *U* might be the result of recent cultural phenomena or a human adaptation specific to music. However, it could also be an adaptation facilitating the cultural transmission of acoustic patterns based on social learning more broadly, which is crucial to music and other non-human communication systems. It thus might also be broadly shared across vocal learners (i.e. species that can modify their vocalizations based on auditory experience), such as birds.

Because the optimal conditions for learning found in many species and the associated preference function found in human music, we hypothesize that, in vocal learners, medium-complexity auditory signals—being sufficiently structured—might be a good compromise to facilitate yet not oversimplify communication and optimize learning. These signals might therefore be preferred over signals of low or high complexity. It is, however, unknown whether birds and other animals exhibit the preference *U* for acoustic patterns.

Here, we thus investigated if preference for intermediate complexity in acoustic patterns is unique to humans or if it is shared with another vocal learning species: the budgerigar (*Melopsittacus undulatus*). Budgerigars are parrots known for their vocal learning abilities and pronounced pattern recognition skills [46–50]. They have been shown to prefer rhythmically regular acoustic patterns over random ones [51] and vocalize using socially transmitted pitch sequences [52,53].

If the inverted U-shaped preference to acoustic stimuli arises from mechanisms and selective pressures shared by vocal learning species or shared more broadly in the animal kingdom, budgerigars should exhibit a preference for intermediate levels of complexity in pitch sequences. Conversely, if this preference is an adaptation specific to music processing and/or the cultural transmission of music, it should be present in humans but not in budgerigars.

We measured the preferences of budgerigars and humans to pitch sequences using a three-option place preference paradigm, with human participants receiving no task instructions to ensure maximum comparability. Both species were presented with the same stimuli. In a given session, listeners could choose between two of the three types of stimuli: either tones arranged randomly, and/or tones arranged into tone patterns that changed every four repetitions, and/or a repetitive tone pattern that always stayed the same. The third option was always silence as the control condition.

## 2. Methods

### 2.1. Stimuli

Stimuli were sequences made from permutations of a set of 10 RMS-normalized 22 ms harmonic tone-pips. Each pip was made of three simultaneous sine tones that represented the fourth to sixth harmonic of a given fundamental frequency. Ten fundamental frequencies were used that were equally spaced on a logarithmic scale between 157.5 and 1250 Hz (12% steps), such that sine 1 = 4f0 | sine 2 = 5f0 | sine 3 = 6f0 (e.g. 630 | 787.5 | 945 for f0 = 157.5; supplementary material, table S1). At 70 dB sound pressure level (SPL), these sounds lie well within the human and budgerigar hearing range [54–56]. All sounds had 3 ms onset and offset amplitude ramps to avoid transients [57,58].

Sequences of the 10 pips were then created such that there was 3 ms of silence between each pip. This was done using three types of permutation procedures corresponding to the three conditions: *random*, *regular* and *regular with changes*. Every sequence consisted of 800 sounds for a total of 20 seconds, and each of the 10 possible pips occurred 80 times. We created five different 20-second sequences for each condition.

For the *random* (Ran) sequences, the 10 pips were concatenated in random order, then all 10 pips were again concatenated in a new random order. Each tone-pip thus occurred with equal probability across the entire sequence.

The *regular* (Reg) sequences consisted of the 10 pips from the set, concatenated in a random order, and then this order was repeated for 80 identical cycles.

For *regular with changes* (VarReg), a pattern of 10 pips was repeated four times, then a different pattern was repeated four times and so on, until 20 different repeated patterns had occurred.

We did not use conspecific-like sounds to prevent socially mediated interference. Budgerigars are highly sensitive to conspecific vocalizations, and pilot observations in our lab suggest that stimuli mimicking budgerigar vocalizations can lead to greater avoidance than synthetic sounds (cf. [59]). To reduce these potential confounds, we used neutral synthetic harmonics distinct from natural vocalizations. Presented as rapid sequences, these stimuli were also unfamiliar to humans. Overall, this approach increased comparability across species while allowing us to more cleanly isolate the effects of stimulus complexity from social or communicative factors.

### 2.2. Information-theoretic modelling

To obtain a quantitative information-theoretic measure of complexity, stimuli were characterized using the prediction by partial matching (PPM)-decay model [60,61], which expresses tone-by-tone unexpectedness in terms of Shannon's information surprise—or information content (IC)—conditioned upon the preceding musical context. PPM-decay ([60], https://github.com/pmcharrison/ppm) is based on a variable-order Markov model framework that learns the stimulus statistics by analysing symbolic sequences event by event. As it processes the sequence, the model stores the occurrences of events and sub-sequences of events (*ngrams*), and it generates a conditional probability distribution for each tone given the preceding tones. Thus, for each event in a sequence, the model quantifies its IC using the probability distribution of previously encountered observations. Contrary to previous learning approaches with perfect memory, PPM-decay also features a number of memory parameters that determine the weighting of historic data, improving its biological plausibility. Here, we used its short-term memory configuration, whereby the model learns only from the current 800-sound sequence. Averaging the IC across all events in a sequence provides an index of the sequence's overall predictability. We interpret this as a measure of complexity, predicting that VarReg sequences (with medium IC) will be perceived as more complex, hence more preferable, than Reg ones (with low IC) and less complex than Ran ones (with high IC).

As shown in figure 1, the model was run on the full stimulus set. A Shapiro–Wilk test suggested that the data were not normally distributed (*p* < 0.001); therefore, a Friedman test was conducted, indicating significant differences between the conditions (*p* = 0.007). Subsequent Wilcoxon tests showed that IC was significantly higher in Ran than in VarReg and Reg (mean IC = 3.5, 1.867 and 0.346, respectively; all Wilcoxon tests, *p* = 0.043), confirming that conditions presented three clearly separable levels of complexity: high, medium and low.

**Figure 1.**
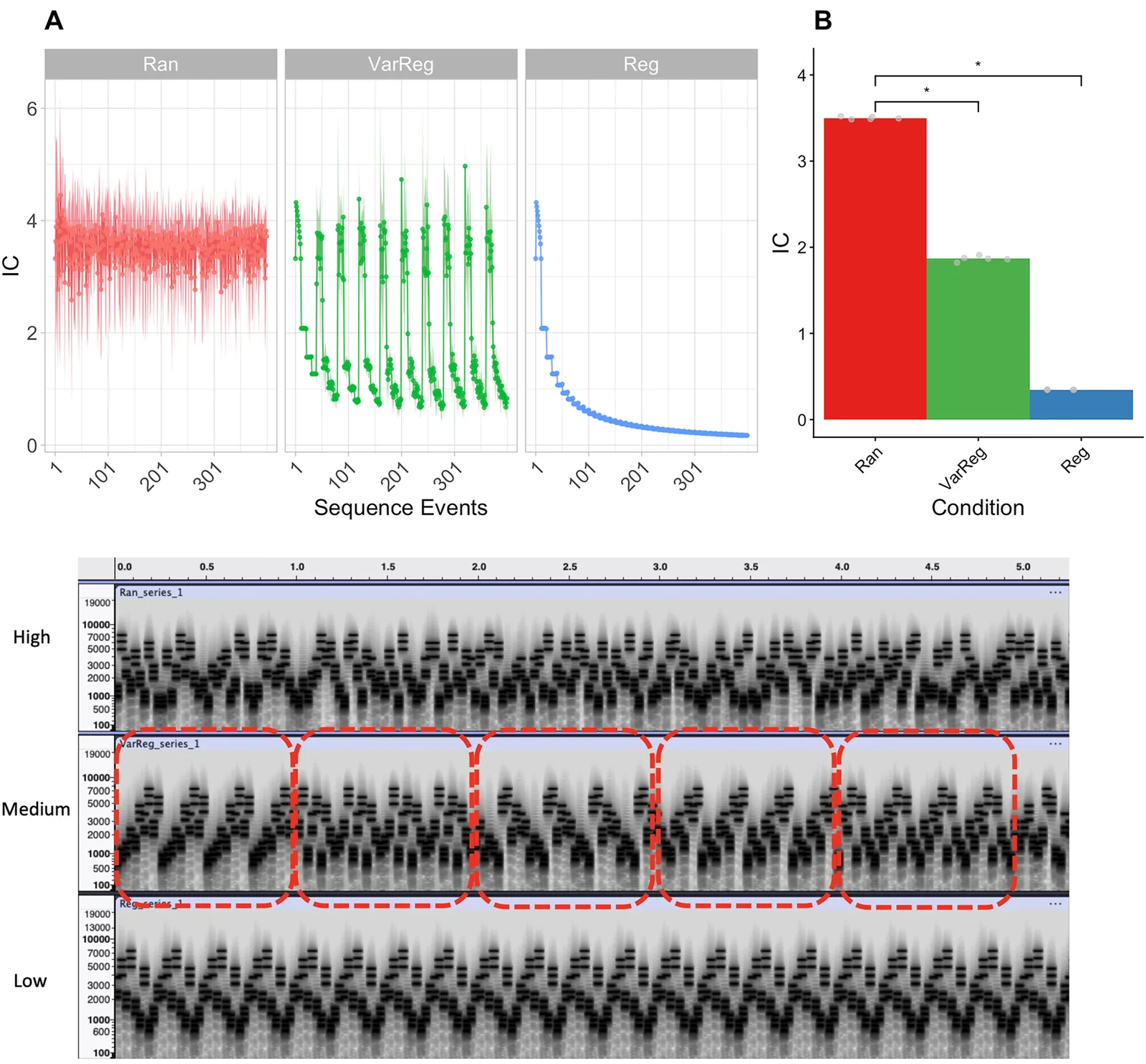
(A) Information content (IC) of the first 400 events (A) and mean IC by condition (B) across the Ran, VarReg and Reg sequences. *Note*: The grey data points in B represent the individual IC values of the sequences. Significance asterisks in B: *p < 0.05. (B) Exemplary spectrograms of Ran (top), VarReg (middle) and Reg (bottom). *Note*: The red areas showcase the patterns consisting of sequences repeated four times. The ordinate represents frequency (in Hz), and abscissa represents time (in seconds).

### 2.3. Participants

We tested *n* = 12 adult budgerigars (six female, six male) housed in the budgerigar lab and *n* = 32 normal-hearing adult humans (15 female, 15 male, one other, one unknown (preferred not to say); mean age = 27.66 years (20–43), s.d. = 4.81 years). Both species were tested at the Acoustics Research Institute of the Austrian Academy of Sciences in Vienna, Austria. Human participants were compensated for participation in the experiments in the form of 10 euros (€) per hour of participation.

### 2.4. Apparatus

We individually assessed the time participants spent with the sequence types as a proxy of preference using a place preference set-up (see [59]).

For budgerigars, this set-up consisted of an aviary with three perches as shown in figure 2A, each fitted with an infrared barrier and adjacent speakers (Vifa full-range speaker 10 BGS 119/8; 104 mm full-range loudspeaker, 80–16 000 Hz frequency range, 86 dB characteristic sound pressure), which were driven by amplifiers adapted from Edirol MA-5D active loudspeaker systems [62]. Sitting on a perch disrupted the infrared barrier, and playback of sound from the adjacent speaker was triggered after greater than 300 ms of disruption (avoiding triggers by fly-throughs). Stimuli were presented at 70 dB SPL at the middle of the perches (digital sound level metre: AZ Instrument, AZ 8922). During every session, a fourth speaker (M-Audio AV40) with an MP3 player (HIFI WALKER MP-7 8 GB) under the testing aviary played a recording from the bird's home aviary at 50 dB to alleviate stress from isolation during testing. Water and food were available at the bottom of the testing aviary ad libitum during sessions.

**Figure 2.**
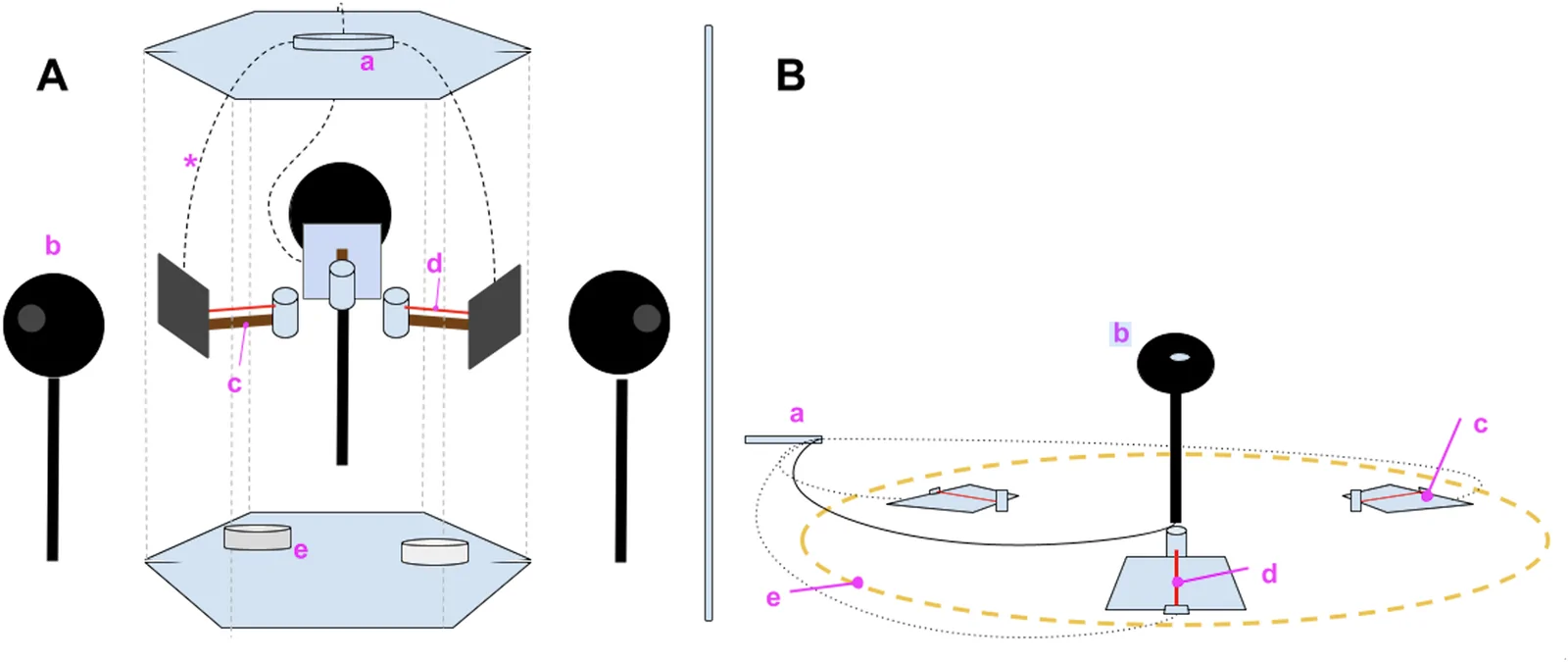
Place preference apparatus for budgerigars (A) and humans (B). *Note*: A: Hexagonal aviary for testing place preference in budgerigars. B: Human place preference arena, a = arduino®; b = speakers; c = perch (A), place preference mats (B); d = infrared beam; e = food and water bowls (A), circular marking on the floor (B).

For humans, the set-up consisted of three floor mats with shoe icons, in a yellow circle (figure 2B). In the circle's centre was a speaker (Vifa full-range speaker 10 BGS 119/8; 104 mm full-range loudspeaker, 80–16 000 Hz frequency range), whose playback level was set to 55 dB SPL at the centre of the mats. Standing on a mat disrupted an infrared beam, which triggered sound playback from the central speaker if the participant stayed for at least 300 ms.

### 2.5. Procedure

For birds, sessions were carried out in the hexagonal aviary (figure 2A; see also [59]). For each 2 hour session, one speaker played one sequence type (e.g. Ran), another played a different sequence type (e.g. VarReg) and the third remained silent. If a listener stayed on a perch (which triggered sound playback) beyond the duration of a stimulus WAV file, a new sequence from the same condition was played. The software cycled through all stimuli from the respective condition in a randomized order until all were played, then repeated the cycle randomly. Leaving the perch would fade out the sound over a 5 ms period; returning to a perch for at least 300 ms triggered the playback of a new stimulus. The program recorded perch condition, time spent on the perch, stimulus playback duration and the number of greater than 300 ms perch visits. Following each session, birds were returned to their home aviary. For each bird, multiple sessions were conducted across different days, such that all of the sequence/perch combinations (e.g. Ran/Reg/Silent) were assessed three times for a total of nine sessions. Across sessions and birds, the positions of sequence types were counterbalanced to minimize effects from perch location biases (supplementary material, table S2).

For humans, the procedure was similar, except that first, the participants were told that their task was to explore the area inside a yellow circle for 5 minutes, that there would be nine such 5 minute sessions, that they could take breaks between sessions and that they could end their participation at any point. Next, participants read and signed a consent form prior to testing. After the sessions, participants completed a listener survey, which included demographic questions (e.g. ‘What is your sex?’). Then, they listened to the sequences heard during the place preference experiment on headphones (Sennheiser HD 280 Pro, 64 Ω) and rated them on predictability, interest and liking, using 7-point visual analogue scales (1 = ‘Not at all’, 7 = ‘Very’). The specific wording used was ‘How much do you like this sequence?’/‘How interesting do you find this sequence?’/‘How predictable (In German: “vorhersehbar”) do you find the sequence?’. Any remaining questions revealing the purpose of the experiments were answered after completion of the surveys.

### 2.6. Data analysis

We analysed the data using R (4.4.1, 2024-06-14) and RStudio (2024.04.2+764, 2024-06-10), employing generalized linear mixed-effects models (glmmTMB package, [63,64]) with a gamma family (see the R script available at OSF). All models included subjects and sessions (as categorical factors) as random effects. We included a dispersion structure (eg. [65,66] and weighting [63,67] to better account for variance. Our primary response variable was the average trial duration per session (mean_trial_time), where a single trial was defined as an individual, continuous, and greater than 300 ms landing on a perch (birds) or standing on a mat (humans) triggering sound playback. We aggregated per session because directly modelling raw data violated model assumptions. Because sessions varied substantially in trial count—the majority of visits lasted less than 500 seconds, while a small proportion exceeded 1 hour—aggregated models were weighted by the number of trials per session. This approach assigns greater influence on session means derived from larger numbers of trials, which represent more precise estimates than means derived from few trials, while also yielding a residual distribution consistent with model assumptions. Condition (Ran/Reg/VarReg/silence) was included as a fixed effect. Because sex differences have been shown in budgerigars' auditory preferences [51] and are currently under investigation for music processing in humans [68], sex was included as an exploratory factor to control for potential variability. Statistical significance was assessed through likelihood-ratio tests conducted using the ‘Anova’ function in the car package [69]. Follow-up contrasts were conducted using the ‘emmeans’ package [70] and the Bonferroni method to account for the increased risk of type I error resulting from multiple comparisons. Model fit was comparatively assessed using the Akaike information criterion and checked using *Q*–*Q* plots and Shapiro-Wilk test. The adjusted *p*-values were calculated to determine significant differences between conditions, with a significance level of *α* = 0.05.

## 3. Results

As shown in figure 3, we investigated how the amount of time spent on the perch per trial differed between the four conditions (Ran, VarReg, Reg, silence). A trial is defined as every instance in which a perch (for birds) or a mat (for humans) was contacted such that the infrared barrier was disrupted for longer than 300 ms. For the relative mean average time the individual budgerigars spent with the different conditions, see supplementary material, fig. S1.

**Figure 3.**
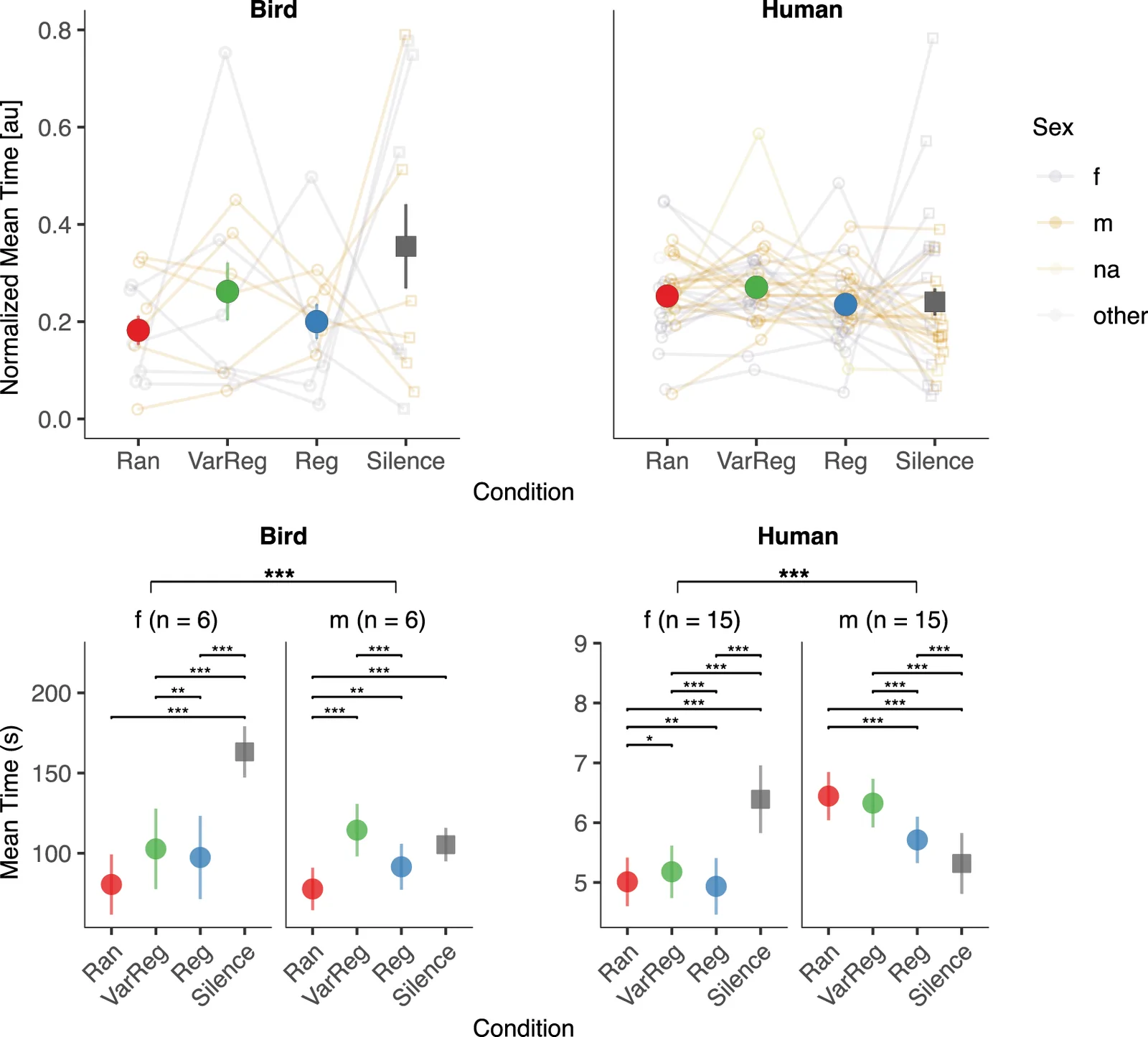
Proportion of mean time per trial across subjects (large dots) and per subject (faint lines) for the four conditions in budgerigars (top left) and humans (top right). The smaller bottom panel shows the mean time per trial for females and males for budgerigars (f & m, left) and humans (f & m, right). *Note*: ordinate: mean time spent in a given condition divided by the sum of mean times across all conditions for that subject (top plot). Mean time per trial in seconds (bottom plots); au = arbitrary unit, abscissa: Ran = random pattern; VarReg = partially repetitive pattern; change of the pattern every four repetitions; Reg = single repetitive tone pattern. For a given session, only three of the four total conditions were available (e.g. Ran, Reg and silence), and silence was always present as a control condition. Significant asterisks reflect the interaction terms (top bars with longer whiskers) and contrasts from the linear mixed models (in-plot bars; *p < 0.05; **p < 0.01; ***p < 0.001). The error bars show the standard error. A figure with the untransformed mean time per trial in seconds for the top plot and detailed sex data are provided in supplementary material, figs. S3 and S4.

The mean times per trial spent per condition were, for the birds: VarReg, 109.46 s; Reg, 93.72 s; Ran, 78.87 s; silence, 129.22 s. For humans, the mean times per trial and condition were VarReg, 6.28 s; Reg, 5.50 s; Ran, 6.12 s; silence, 5.86 s.

For birds, the model (formula: ∼ condition * sex + (1 | subject) + (1 | session), dispformula = ∼ condition, family = gamma(link = ‘log’) revealed a significant main effect of condition ($\chi^{2}$(3) = 269.79, *p* < 0.001), but not sex ($\chi^{2}$(1) = 0.06, *p* = 0.806). The interaction between condition and sex was significant ($\chi^{2}$(3) = 188.52, *p* < 0.001).

A post hoc analysis with pairwise comparisons showed that the birds spent significantly more time with VarReg than with Reg (*p* < 0.001) and Ran (*p* = 0.013) but less than with silence (*p* < 0.001). The other contrasts also yielded significant differences (silence > Ran > Reg, silence > Reg, all *p* < 0.001).

Regarding the interaction of sex and condition (figure 1, bottom panel), contrasts per sex revealed that for females, the time spent with VarReg was significantly greater than for Reg (*β* = 0.229, *p* = 0.004), yet only marginally greater than for Ran (*β* = 0.167, *p* = 0.081). There was no significant difference between Ran and Reg (*β* = 0.0627, *p* = 1). The time spent with silence was significantly greater than all other conditions (Reg, Ran and VarReg, all *p* < 0.001).

Males spent more time with VarReg compared to Ran (*β* = 0.388, *p* < 0.001) and Reg (*β* = 0.221, *p* < 0.001), as well as with Reg versus Ran (*β* = 0.168, *p* = 0.003). The time spent on silence was greater compared to Ran (*β* = 0.247, *p* < 0.001), but not compared to VarReg (*β* = 0.141, *p* = 0.235) and Reg (*β* = 0.079, *p* = 1).

For humans, the model (formula: time ∼ condition * sex + (1 | subject) + (1 | session), dispformula = ∼ condition + sex + session, family = gamma(link = ‘log’) revealed a significant main effect of condition ($\chi^{2}$(3) = 154.81, *p* < 0.001) but not for sex ($\chi^{2}$(2) = 3.08, *p* = 0.214). The interaction between sex and condition was significant ($\chi^{2}$(6) = 318.91, *p* < 0.001).

Post hoc pairwise comparisons showed that human participants spent significantly more time with VarReg than with the other conditions (Reg: *β* = 0.505, *p* < 0.001; Ran: *β* = 0.222, *p* < 0.001; silence: *β* = 0.400, *p* < 0.001). The time spent with Ran was significantly higher than with Reg (*β* = 0.283, *p* < 0.001) and silence (*β* = 0.178, *p* = 0.003). There was no significant difference between Reg and silence (*β* = −0.105, *p* = 0.164).

Regarding the interaction of sex and condition, post hoc contrasts revealed that females spent significantly more time with VarReg than with Reg (*β* = 0.187, *p* < 0.001) and Ran (*β* = 0.078, *p* = 0.041), as well as with Ran compared to Reg (*β* = 0.108, *p* = 0.001). The time on silence was significantly greater compared to all conditions (Ran: *β* = 0.479, Reg: *β* = 0.587 and VarReg: *β* = 0.400; all contrasts *p* < 0.001).

Males, similarly, spent more time with VarReg than with Reg (*β* = 0.111, *p* < 0.001) and with Ran compared to Reg (*β* = 0.1009, *p* < 0.001), while there was no difference between VarReg and Ran (*β* = 0.010, *p* = 1). Contrasting the female pattern, males spent significantly less time with silence than with all other conditions (Ran: *β* = 0.256, Reg: *β* = 0.155 and VarReg: *β* = 0.265; all contrasts *p* < 0.001).

Figure 4 shows the listener survey results.

**Figure 4.**
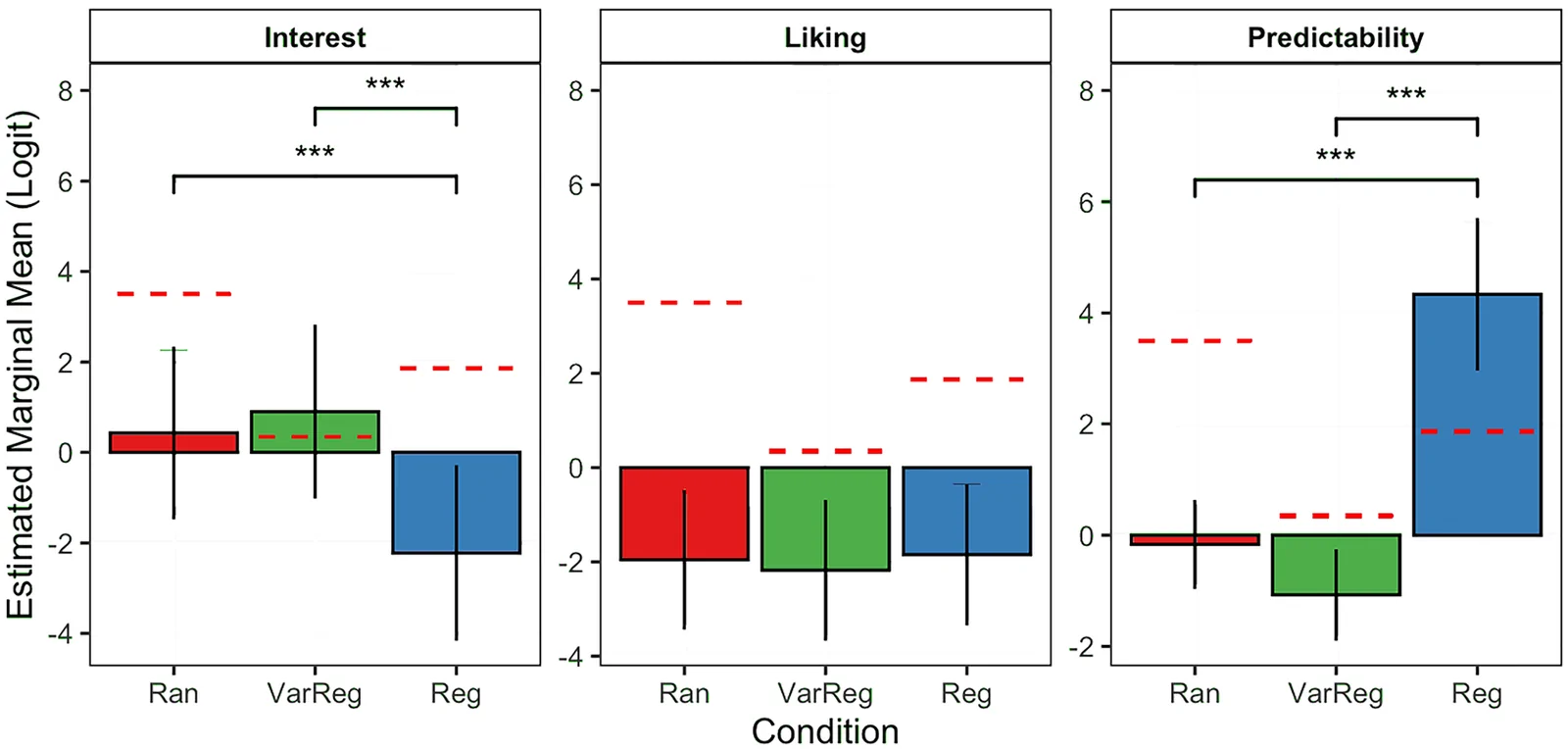
Listener survey results: estimated marginal means of the ratings per condition (abscissa) and standard error across subjects. *Note*: Red dashed lines indicate the IC values. See supplementary material, fig. S2, for individual ratings. *** contrast: *p* < 0.001.

Concerning predictability, cumulative link mixed-effects models (formula: rating ordinal ∼ condition + sex + (1 | subject)) showed significant main effects of the sequence type ($\chi^{2}$(2) = 39.92, *p* < 0.001), no effect of sex (*p* = 0.246). A post hoc analysis with Bonferroni-corrected pairwise contrasts revealed that predictability was greater for Reg than VarReg (*p* < 0.001) and Ran (*p* < 0.001), while there was no difference between Ran and VarReg (*p* = 0.175).

In the model for interest, there was a main effect of the sequence type ($\chi^{2}$(2) = 35.18, *p* < 0.001) and sex ($\chi^{2}$(3) = 12.08, *p* = 0.007). Pairwise contrasts revealed that compared to Reg, interest ratings were greater for VarReg (*p* < 0.001), as well as for Ran (*p* < 0.001), while there was no difference between VarReg and Ran (*p* = 0.855).

In the model for liking, there was no effect of sequence type ($\chi^{2}$(2) = 0.40, *p* = 0.817) and no effect of sex ($\chi^{2}$(1) = 3.40, *p* = 0.065).

## 4. Discussion

We investigated the relationship between stimulus complexity and preference in the auditory domain across two vocal learning species: budgerigars and humans. This relationship was generally proposed to be shaped by mechanisms that preferably draw organisms to intermediate complex information optimal for learning. We investigated whether such an inverted U-shape relationship (the *U*) applies to the auditory domain in vocal learners, because their communication greatly depends on efficient learning in the auditory domain. Using pitch sequences of low, intermediate and high complexity, we tested preferences in budgerigars and humans, two vocal learning species, each with communication systems that rely on socially transmitted pitch sequences.

Our principal finding is that both budgerigar and human preference for pitch sequences exhibit a nonlinear (intermediate) preference profile. Although the categorical nature of the design (i.e. the three discrete levels of complexity) limits strong claims about the exact functional form, the pattern is consistent with an inverted U-shape relationship. Despite likely differences in temporal resolution (Dooling *et al.*, 2002 [71])—budgerigars may better resolve our rapid sequences than humans—both species exhibited a U-shaped response. This suggests that the U-shaped relationship may be a shared, general principle of auditory information processing in these two species rather than exclusively holding for a specific granularity of the input. The similarity of our results across two genetically distant species of vocal learners could possibly reflect behaviour guided by a similar optimized learning strategy for music and bird communication, two widely recognized model systems for vocal learning (e.g. [14,72]). This potential strategy may guide learning by channelling cognitive resources towards auditory material based on its statistical properties. Similar to the visual domain [6], it may prioritize material of medium complexity that balances novelty and familiarity: complex enough to offer new information and build new models of the world but also sufficiently related to already existing models to enable their consolidation and refinement. Learners have to deploy their limited resources optimally, because it is impossible to use all available information. Similarly, ignoring opportunities for learning hampers the improvement and expansion of existing skills and knowledge [6,73]. The suggestion that there are mechanisms guiding animals to stimuli useful for learning is supported by evidence from an investigation of *in ovo* sound processing in birds. Compared to vocal non-learning species, vocal learners show stronger physiological responses to conspecific vocalizations (i.e. increases in heart rate) than vocal non-learning species already *in ovo* [74]. This heightened response strength suggests that vocal learners, compared to non-learners, may have a broader and/or additional set of mechanisms that first guide attention to important stimulus types before filtering them according to complexity.

However, because we did not test vocal non-learners, it is still unknown whether the U-shape preference for sounds is also present in these species. In general, vertebrates show an inverted U-shape relationship in a variety of behavioural, non-auditory contexts, for example, between prey size and preference of prey choice [75], the familiarity of playmates [76] or the level of surprise in sequences of visual shapes [6]. Concerning the auditory domain, research in vocal non-learners, such as mice, showed that moderate states of arousal are associated with better performance for both categorizing polyphonic melodies in a discrimination task [77] and for a tone-in-noise detection task [78]. Further testing is needed to reveal whether a U-shaped learning strategy in the auditory domain is shared beyond vocal learners and also whether preference for mid complex sounds is found in more species. These avenues for research into auditory cognition across the animal kingdom would be essential to scrutinize the potential links between the inverted U-shape and vocal learning.

The overall attraction of birds and humans to patterns that are probably better for learning indicates links of *the U* to aesthetic phenomena. In general, stimuli that are more appealing may drive animal behaviour for a variety of functional goals, often linked to social learning, such as mate choice [79] and nest material selection [80]. *The U* we observed here may be a driving force underlying aesthetic cognition. Aesthetic cognition, the capacity to perceive and create beauty, as well as making choices based on aesthetic judgements, is potentially shared across many species [81–83]. Our experiment, being designed for cross-species use, could be used for large-scale cross-species investigations, pushing that line of inquiry by further examining the relationships between complex auditory stimuli and preference.

Despite the differences between the sequence types, the most time in budgerigars was spent with the silent condition. First, since budgerigars are a species that show strong neophobic tendencies [84], this overall avoidance may be grounded in the unfamiliarity of the sounds to the birds. Second, the general preference for pitch sequences over silence in humans, but not in budgerigars, may be rooted in motivational ties to music unique to humans, mirroring similar findings comparing macaques and humans [85].

Listener sex appeared to influence responses to the silent condition, as analysis of sex differences revealed parallels in preferences across both budgerigars and humans (figure 3, bottom panels). Across both species, females preferred silence over all stimuli. By contrast, this preference was overall absent in males. In budgerigars, males preferred silence only when compared to Ran; in humans, males spent significantly more time with all auditory stimuli than with silence. The drivers of these sex-specific profiles remain speculative. It has been suggested that, in humans, males may exhibit higher levels of novelty-seeking or a greater susceptibility to boredom in place preference tasks, potentially leading to increased exploration [59]. This aligns with reports that male budgerigars show greater inclination towards atypical stimuli [51] and less avoidance behaviour towards alarm calls compared to females [86]. Anyhow, evidence for a universal sex difference in novelty-seeking remains inconclusive (e.g. [87]). Although our results suggest a similar behavioural pattern where females prioritize silence and males prioritize stimulation, further research is required to determine whether these preferences are rooted in differences in risk-aversion, sensory processing or exploratory drive.

A few things about our human results are of note. We observed a discrepancy between behavioural place preference and aesthetic ratings, as ‘liking’ scores did not differ across conditions, and ‘interest’ scores only partially supported the inverted U. Similar discrepancies between listening time and explicit ratings have been reported in previous research [88]. Notably, preference is not a monolithic concept. As established in addiction research, ‘incentive wanting’ and ‘hedonic liking’ are distinct systems [89,90]. Our place preference task probably captures an appetitive–aversive axis, whereas the survey asks for a cognitive evaluation of hedonic value (e.g. ‘liking’—a flat line here). This might mirror the experiences of challenging art, minimal music or horror cinema, where stimuli may be appetitive without being traditionally appealing. Thus, while our stimuli elicited a behavioural preference for intermediate complexity, this effect was not consistently reflected in explicit ratings, suggesting a partial dissociation between behavioural and evaluative measures.

Also, our additional sound rating task with humans revealed that high-complexity random sequences were rated as more predictable than our medium-complexity sequences that repeated and then changed, despite the higher IC of high-complexity (Ran) sequences. High-complexity sequences were potentially characterized by being predictably unpredictable, a phenomenon that can lead to low uncertainty despite fluctuating information [91]. To illustrate this with an example, the individual sounds of the drops that constitute the event type ‘rain’ are impossible to time, yet the sound of rain itself is probably not perceived as unpredictable, but rather soothing and constant. By contrast, with the medium-complexity (VarReg) sequences, only the pattern change itself was predictable, occurring after every four repetitions. The contents of the patterns (i.e. both their contour and the order of individual tone-pips), however, were randomized anew with every change of the pattern, possibly leading to a perceived uncertainty higher than that of the high-complexity sequences.

Notably, interest and predictability ratings mirrored each other. In other words, medium complex stimuli were rated the most interesting and the least predictable. Conversely, low-complexity stimuli were rated the least interesting, while high-complexity stimuli obtained intermediate ratings in both interest and predictability.

Previous research has highlighted that the relationship between aesthetic judgements and predictability is complex and dependent on the immediate context; auditory stimuli are situated in [92]. Rather than preferring information that is predictable, humans prefer information that is predictive of other events in the immediate context [93]. This suggests that people find stimuli that are predictive of other events more interesting or attention-worthy than stimuli that are predictable but are not predictive of other events. In our stimuli, the internal structure of the medium-complexity sequences and their predictiveness might explain the higher interest ratings for these sequences. Four repetitions of a randomly assembled tone sequence would trigger the start of a new random sequence to be repeated four times and so on. The medium-complexity stimuli thus can be regarded as a succession of events that, like a countdown, indicated the impending change of event to a new sequence, which again clocked down four times to again trigger the event of a change in pattern to a new sequence. Therefore, the medium-complexity sequences were rather unpredictable in terms of their content, yet their subordinate events were highly predictive of other events, that is, the change of pattern.

Taken together, preferences for predictive information may explain the complementarity of the U-shaped predictability ratings and the inverted U-shaped interest ratings, while also accounting for the human and budgerigar results from the place preference apparatus. Because preference for predictive stimuli is found in non-human primates [94,95], pigeons [96] and rats [97], future work with targeted, power-informed designs will be important to replicate and extend these findings. Overall, our results suggest that investigating the propensity for predictive stimuli, in conjunction with preference for intermediately complex stimuli across a wider range of animals, might be an exciting avenue for research into the foundations of aesthetic perception across the animal kingdom.

## Supplementary Material



## Data Availability

The datasets for this study can be found in the OSF repository [98]. Supplementary material is available online [99].
