# Supplementary material for: Preferences for pitch sequence complexity are consistent with an inverted U-shape in humans and parrots
Source: R Soc Open Sci. 2026 Oct 21;13(10):252399. doi: 10.1098/rsos.252399 (PMC13682898; doi:10.1098/rsos.252399)
Supplement: Supplementary file 1 [file rsos.252399.zip › rsos.252399/rsos252399_si_001.docx]

## Supplementary materials

**Supplementary Table 1**

*Sine triads used in the pitch sequences.*

| **Triad no.** | **f sine 1 (Hz)** | **f sine 2 (Hz)** | **f sine 3 (Hz)** | **virtual f0** |
| --- | --- | --- | --- | --- |
| **1** | 630 | 787.5 | 945 | 157.5 |
| **2** | 794 | 992.5 | 1191 | 198.5 |
| **3** | 1000 | 1250 | 1500 | 250 |
| **4** | 1260 | 1575 | 1890 | 315 |
| **5** | 1588 | 1985 | 2382 | 397 |
| **6** | 2000 | 2500 | 3000 | 500 |
| **7** | 2518 | 2147.5 | 3777 | 629.5 |
| **8** | 3174 | 3967.5 | 4761 | 793.5 |
| **9** | 4000 | 5000 | 6000 | 1000 |
| **10** | 5000 | 6250 | 7500 | 1250 |

Note: f = frequency. f0 = fundamental frequency, i.e., the basis of the harmonic series of the triads’ individual frequencies. Hz = Hertz.

**Supplementary Table 2**

*Scheme for counterbalancing stimuli positions across sessions and participants.*

| **Participant** | **Session** | **Pair** | **Position 1** | **Position 2** | **Position 3** |
| --- | --- | --- | --- | --- | --- |
| *Bird A* | 1 | Ran vs VarReg | Silent | VarReg | Ran |
|  | 2 | Ran vs Reg | Reg | Ran | Silent |
|  | 3 | Reg vs VarReg | VarReg | Silent | Reg |
|  | 4 | Ran vs VarReg | Silent | Ran | VarReg |
|  | 5 | Ran vs Reg | Silent | Reg | Ran |
|  | 6 | Reg vs VarReg | Reg | Silent | VarReg |
|  | 7 | Ran vs VarReg | VarReg | Silent | Ran |
|  | 8 | Ran vs Reg | Reg | Ran | Silent |
|  | 9 | Reg vs VarReg | Silent | VarReg | Reg |
|  |  |  |  |  |  |
| *Bird B* | 1 | Ran vs Reg | Reg | Ran | Silent |
|  | 2 | Ran vs VarReg | Reg | Silent | VarReg |
|  | 3 | Reg vs VarReg | Silent | Reg | VarReg |
|  | [...] | [...] | [...] | [...] | [...] |

**Supplementary Figure 1**

*Individual relative mean average time the budgerigars spent with the different conditions.*

Note. Divided by the gap: Left females, right males.

**Supplementary Figure 2**

*Mean Ratings per Subject and Overall Mean*

Note. Points represent individual subject means; Boxplots show median and IQR.

**Supplementary Figure 3**

*Mean Time per Trial per Sex and Species*

**Supplementary Figure 4**

*Proportion of Mean Time Per Trial with Overall Means only, per Condition.*

Note. The Y-axis shows the mean time spent in a given condition divided by the sum of mean times across all conditions for that subject
